# Pursuit and escape drive fine-scale movement variation during migration in a temperate alpine ungulate

**DOI:** 10.1038/s41598-024-65948-8

**Published:** 2024-07-02

**Authors:** Christian John, Tal Avgar, Karl Rittger, Justine A. Smith, Logan W. Stephenson, Thomas R. Stephenson, Eric Post

**Affiliations:** 1 Department of Wildlife, Fish, and Conservation Biology, University of California, Davis, Davis, CA USA; 2 Marine Science Institute, University of California, Santa Barbara, Santa Barbara, CA USA; 3 Department of Wildland Resources and Ecology Center, Utah State University, Logan, UT USA; 4 Department of Biology, University of British Columbia - Okanagan, Kelowna, BC Canada; 5 Wildlife Science Centre, Biodiversity Pathways Ltd., Kelowna, BC Canada; 6 Institute of Arctic and Alpine Research, University of Colorado, Boulder, Boulder, CO USA; 7 California Department of Fish and Wildlife, Sierra Nevada Bighorn Sheep Recovery Program, Bishop, CA USA

**Keywords:** Altitudinal migration, Bighorn sheep, Elevational migration, Endangered species, Green wave hypothesis, Migration phenology, iSSA, Animal migration, Climate-change ecology, Phenology

## Abstract

Climate change reduces snowpack, advances snowmelt phenology, drives summer warming, alters growing season precipitation regimes, and consequently modifies vegetation phenology in mountain systems. Elevational migrants track spatial variation in seasonal plant growth by moving between ranges at different elevations during spring, so climate-driven vegetation change may disrupt historic benefits of migration. Elevational migrants can furthermore cope with short-term environmental variability by undertaking brief vertical movements to refugia when sudden adverse conditions arise. We uncover drivers of fine-scale vertical movement variation during upland migration in an endangered alpine specialist, Sierra Nevada bighorn sheep (*Ovis canadensis sierrae*) using a 20-year study of GPS collar data collected from 311 unique individuals. We used integrated step-selection analysis to determine factors that promote vertical movements and drive selection of destinations following vertical movements. Our results reveal that relatively high temperatures consistently drive uphill movements, while precipitation likely drives downhill movements. Furthermore, bighorn select destinations at their peak annual biomass and maximal time since snowmelt. These results indicate that although Sierra Nevada bighorn sheep seek out foraging opportunities related to landscape phenology, they compensate for short-term environmental stressors by undertaking brief up- and downslope vertical movements. Migrants may therefore be impacted by future warming and increased storm frequency or intensity, with shifts in annual migration timing, and fine-scale vertical movement responses to environmental variability.

## Introduction

Recent and ongoing climate change disrupt the spatiotemporal pattern of spring plant growth in temperate regions through modified precipitation and temperature regimes^1–3^. Because ungulates commonly track plant phenology during their spring migration^4–6^, climate change may affect spatiotemporal patterns of herbivore movement and migration^7^. Forage tracking is a useful tactic for ungulates in landscapes with gradients in plant phenology, because access to highly digestible plant material is maintained or maximized through time^8,9^.

Many migratory ungulates track forage phenology across elevational gradients in a form of seasonal vertical migration^10–12^. Although vertical migration in ungulates may emerge in a traditional, “undistracted” form of movement from one range to another, the geographical proximity of seasonal ranges separated by elevation allows migrants to use a broader portfolio of migration strategies^13^. By doubling back over migratory corridors, short-distance elevational migrants may undertake multiple up- and down-slope movements in response to environmental variability that could not be practically carried out in a long-distance migration. Elevational migrants may therefore undergo several sub-seasonal movements during a foraging season to maximize resource access across multiple seasonal ranges^14,15^.

Fine-scale movements during the migratory season could, however, be additionally influenced by factors other than foraging opportunities. Because landscapes of relief generate variation across multiple ecological and climatic axes, vertical movements enable herbivores to respond to change in multiple environmental conditions^16^. Vertical movements may allow migrants to alleviate or intensify realized environmental conditions through both static landscape variation (ecological variability across space but not time) and dynamic landscape variation (variability across both space and time). Whereas seasonal variation in snow cover and forage availability may ultimately underlie seasonal redistribution of migrants, variation in exposure to high temperatures or severe storms can be mitigated by moving across elevation at daily or hourly scales^17,18^. Because temperatures tend to decrease at higher elevations, upward movements can lead to a reduction in experienced heat; conversely, dangers associated with precipitation and storms on alpine plateaus can be relieved by moving downslope into comparatively protected canyons^19^.

**Figure 1:**
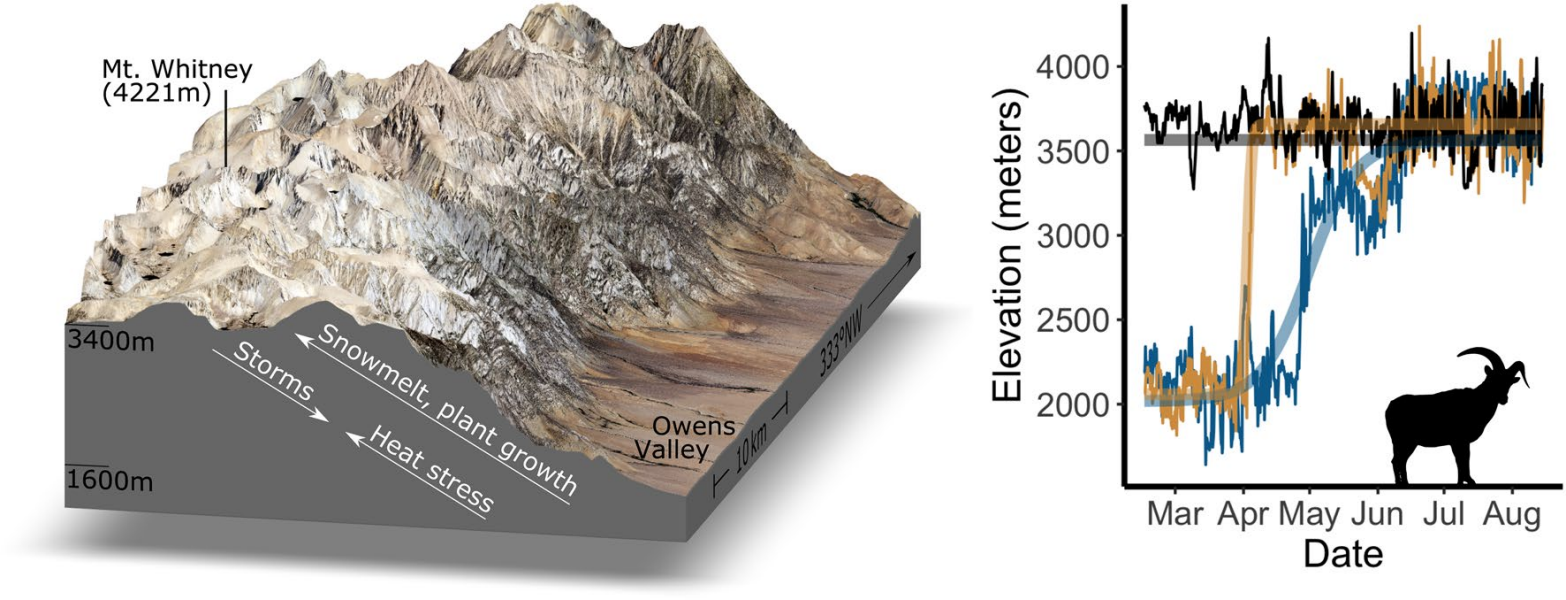
Sierra Nevada bighorn sheep confront variation in stressors and resources throughout spring migrations between low-elevation winter range and high-elevation summer range (left). For some individuals, spring migration follows a unidirectional, undistracted path (right, tan), whereas for others multiple up-and-down movements slow down the mean pace of vertical redistribution (blue). For yet other individuals, wintertime residency at high elevations leads to consistent use of a narrower range of elevational strata throughout the spring (black).

The objective of this study was to evaluate the extent to which static and dynamic variation in environmental conditions leads to complex use of elevation in an herbivorous elevational migrant, Sierra Nevada bighorn sheep (“Sierra bighorn”, *Ovis canadensis sierrae*). Sierra bighorn is a federally endangered subspecies of bighorn sheep endemic to the Sierra Nevada mountains of California (USA) that migrate between the Owens Valley (1500–2000 m a.s.l.) and the High Sierra (3000–4000 m a.s.l.) each spring, but with substantial variation in day-to-day elevation use^13^ (Fig. 1). We expected that spring migration timing and habitat selection would broadly correspond with landscape phenology, but that fine-scale variation in elevation use during upland migration would arise in response to fine-scale stressors such as high temperature (which may lead to heat stress) and precipitation events (which may increase risk of terrain instability and rockfall). We furthermore expected that slow migrants would exhibit more elevational response to short-term environmental variability than fast migrants or residents, which are more constrained to particular elevational ranges. Similarly, we expected that ewes would be more restricted to high-elevation habitat, because refuge from low-elevation predators improves reproductive success during the coincidental lamb rearing season. To test these expectations, we used a three-part approach to explore Sierra bighorn movement responses to dynamic landscape variation: First, we determined whether upslope migration timing was related to snowmelt and green-up timing. Second, we tested the extent to which variation in environmental stressors and resources promoted adjustments in elevation use. And third, we evaluated how selection for terrain features and weather, snow, and forage conditions differed by sex, migratory status, and migratory strategy.

## Results

**Figure 2:**
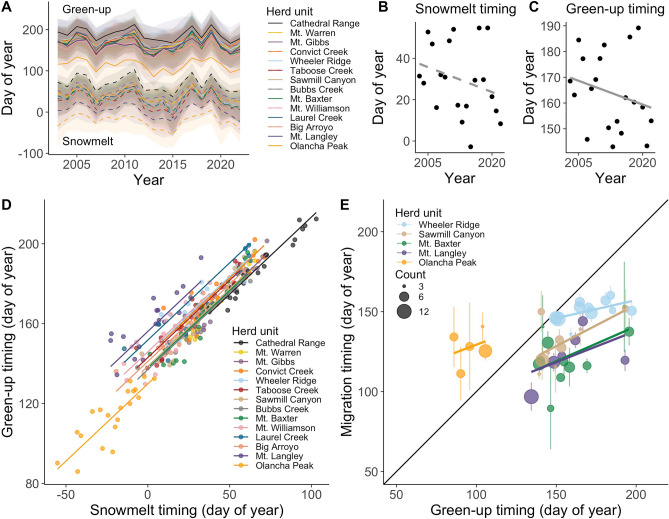
Snowmelt timing (dashed lines) and subsequent green-up timing (solid lines) varied by herd unit and year across the range of Sierra Nevada bighorn sheep, 2003–2022 (**A**). Across the full time series, a significant trend toward earlier dates was evident for both mean snowmelt timing (**B**) and green-up timing (**C**). Mean green-up timing was significantly associated with snowmelt timing within the herd unit level (**D**). Mean migration timing vs. green-up timing for herd units with at least 3 individuals tracked in at least 5 years (**E**; migration timing points scaled by number of individuals tracked and bars are 1 standard error).

Green-up and snowmelt timing varied from year to year but both advanced significantly over the course of the study (Fig. 2A–C). A linear mixed-effects model with snowmelt or green-up timing as the response variable, year as the predictor, and herd unit as a random effect, revealed that snowmelt timing advanced at a rate of 8.03 ± 1.80 days per decade, and green-up timing advanced at a rate of 6.14 ± 1.57 days per decade (*p* < 0.001 in both cases). Variability within herd units (indexed as the standard deviation of green-up or snowmelt timing each year) did not significantly change over the course of the time series (*p* > 0.05).

Across the study system, green-up timing (as measured at the herd unit level) was consistently later in years when snowmelt timing (as measured at the herd unit level) was later (Fig. 2D). In a mixed-effects model with green-up timing as the response variable, snowmelt timing as the predictor variable, and year as a random intercept, green-up was 7.7 ± 0.15 days later per 10-day delay in snowmelt (*p* < 0.001; conditional R^2^ = 0.92). Thus, years with especially early snowmelt were characterized by a greater lag between the snowmelt timing and green-up timing. The mean difference in timing between peak snowmelt and peak green-up was 136.7 ± 0.86 days.

Uphill migration timing covaried with green-up timing, and in years with later green-up timing, migration timing was delayed as well (Fig. 2E). Mean migration timing occurred before mean green-up timing at the herd unit level in 75.2% of cases; however, anomalously late migrations were observed in several cases when bighorn undertook out-of-season vertical movements. The earliest migration (relative to green-up timing) occurred 124 days prior to mean green-up timing (at the herd unit level), while the latest was 79 days after mean green-up timing. A linear mixed-effects model with migration timing as the response variable, green-up timing and sex as predictors, and herd unit identity and year as random intercepts, revealed that the midpoint of migration was 4.1 ± 1.1 days later per 10-day delay in green-up timing (*p* = 0.001; conditional R^2^ = 0.46). Migration was 5.9 ± 3.5 days earlier for females than males, but this difference was not significant (*p* = 0.09).

**Figure 3:**
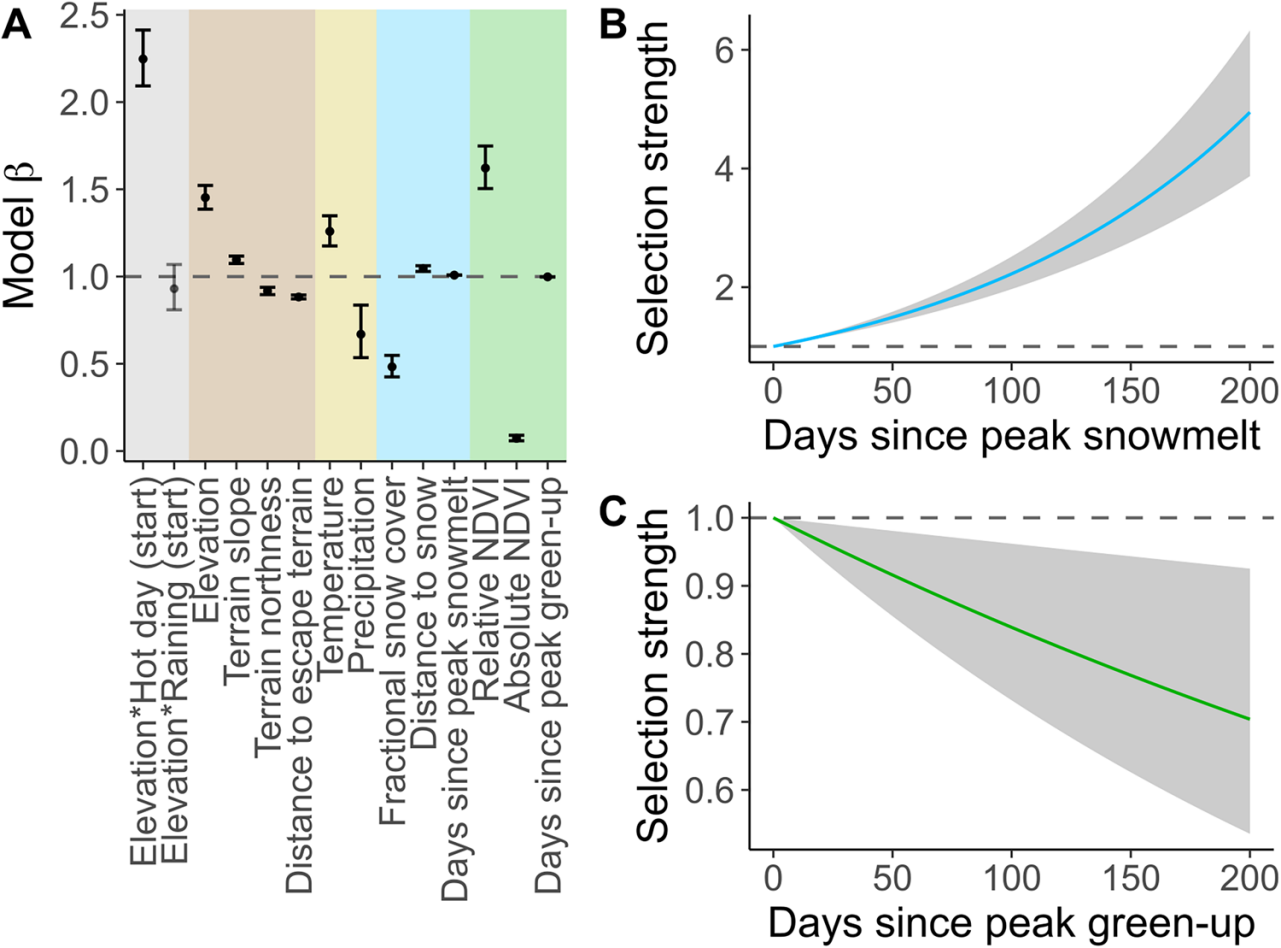
Coefficients from population-level integrated step-selection analysis of Sierra bighorn movement (**A**). Terrain parameters shown in brown, weather parameters in beige, snow parameters in blue, and forage parameters in green. Interactions between startpoint conditions (the binary statuses of hot day and raining) and endpoint elevation shown in grey. Selection strength increased for destinations with increasing days since peak snowmelt relative to destinations where snowmelt occurred 0 days ago (**B**). Conversely, selection strength decreased for destinations with increasing days since peak green-up relative to destinations where green-up peaked 0 days ago (**C**).

Throughout the spring migratory season, bighorn selected steep, south-facing slopes close to escape terrain (Fig. 3A). Bighorn selected warmer areas and avoided precipitation. A strong positive interaction between high temperature at the start of movement and endpoint elevation indicates that bighorn were especially likely to select uphill steps on hot days (Elevation*Hot day (start)). They generally avoided snow, selecting habitat with low fractional snow cover, away from dense snowpack, and that had been snow-free for longer (Fig. 3B). Bighorn avoided moving toward areas with large absolute NDVI (i.e. sites with high overall plant biomass) and instead preferred areas with high relative NDVI (i.e. sites that were near their local greenness maxima for the year), and sites that were at or near their peak rate of green-up (Fig. 3C). Comparison by QIC (a quasi-likelihood weighted generalized measure for AIC^20^) among models fit with all predictors and nested models fit with only terrain, weather, snow, or forage parameters revealed the most support for the overall model (∆QIC > 500 for all nested models).

**Figure 4:**
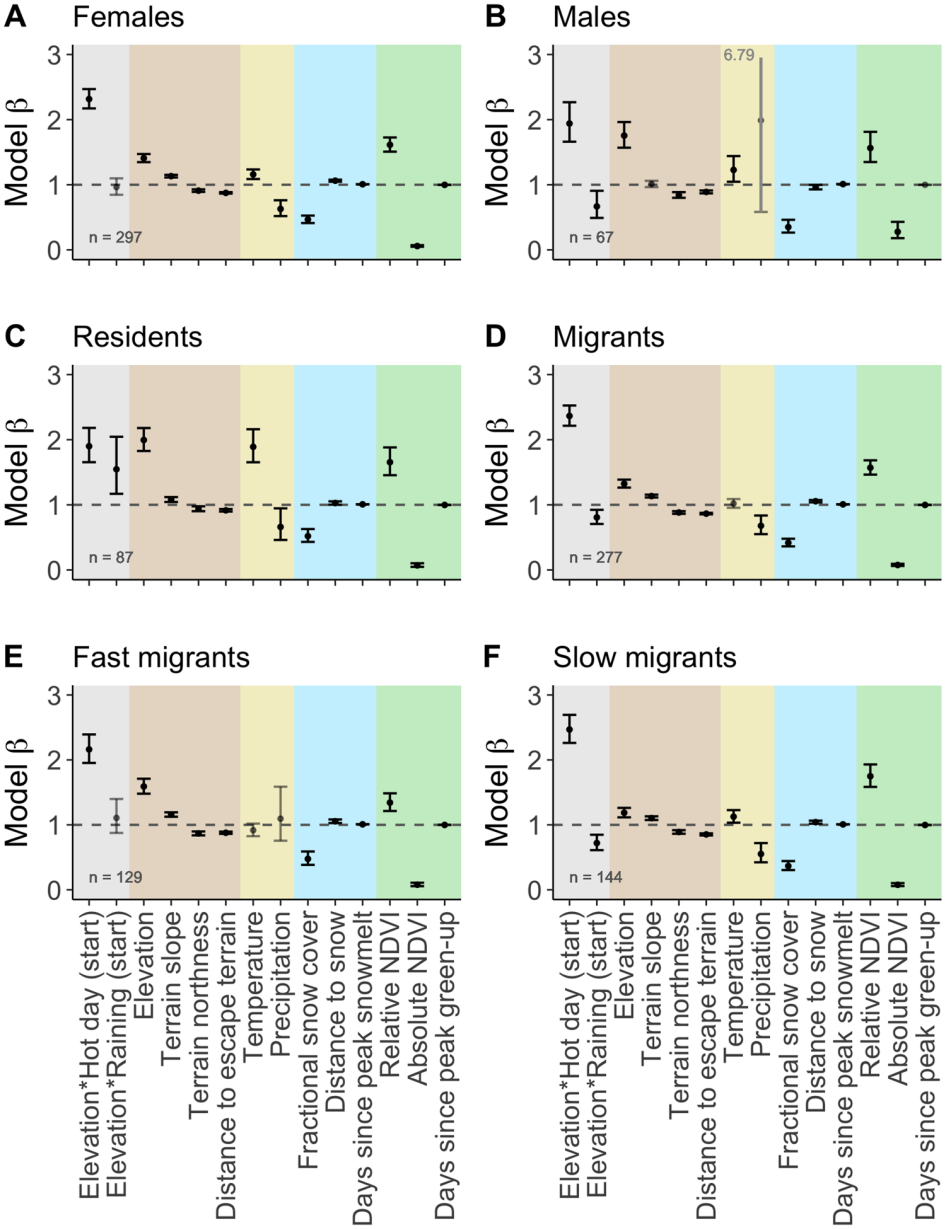
Model coefficients (exponential estimated parameter ± S.E.) from population movement subsets for females (**A**), males (**B**), residents (**C**), migrants (**D**), fast migrants (**E**), and slow migrants (**F**). Inset “n” refers to the number of animal-years represented in the population subset model. Note that in (**B**), one error bar exceeds axis limits and its terminus is indicated numerically.

Selection for high-elevation, steep, south-facing slopes that were close to escape terrain was consistently evident across population subsets (Fig. 4A–F; for summary of classes by herd unit and year, see Supplementary materials S3). A positive interaction between high temperature at the start of movement and endpoint elevation (Elevation*Hot day (start)) was also detected for all groups. Patterns of selection in response to snow and forage parameters were generally conserved across groups. A negative interaction between rain at the start of movement and endpoint elevation (Elevation*Raining (start)) indicated that males, migrants, and in particular slow migrants selected against high elevations on rainy days. The interaction was positive or neutral for females, residents, and fast migrants.

## Discussion

Sierra Nevada bighorn sheep undertake a partial, facultative vertical migration during the spring snowmelt and green-up season^21^, but their vertical movements are rarely unidirectional and often lead to complex use of elevation^13^. Although seasonal variation in space use leads to a general pattern of redistribution across elevation, our results indicate that fine-scale vertical movements during the migratory season might allow Sierra bighorn to realize multiple goals, including pursuing foraging opportunities, avoiding heat stress, and seeking refuge from storms. Because the seasonal ranges of Sierra bighorn are separated by a day’s walk or less, across 1–2 km elevation^22^, individuals can reverse their migration with little consequence for geographic proximity to optimal foraging opportunities. This differs from long-distance migratory ungulates such as mule deer^12^ and caribou^23^, for which retreat along the migratory route might complicate access to high-quality resources^9^.

Uphill migration timing by Sierra bighorn was broadly associated with green-up timing at the herd unit level, which was in turn associated with snowmelt timing. Coordinating migration timing with resource phenology is common among ungulates, because foraging efficiency increases with access to highly digestible early-stage plant growth^24–26^. The Sierra Nevada mountains feature strong interannual variation in snow cover, snowmelt timing, and green-up timing, with over 3 months between the earliest and latest green-up records over the course of this study. Accordingly, we observed a high rate of switching between migration and residency by individuals among years (mean strategy switch rate = 0.46 ± 0.04) and switching between fast and slow migration tactics among years (mean rate switch rate = 0.62 ± 0.06). Flexible migratory behavior in ungulates may increase foraging opportunities, but can also alleviate intraspecific competition, reduce predation risk, and reduce pathogen exposure^27^.

Our full step selection model identified strong associations between terrain, weather, snow, and forage factors in driving step selection. Furthermore, the terrain-only model was the best performing nested step selection model. Among the group models (i.e. females, males, residents, migrants, fast migrants, and slow migrants), terrain factors were consistently important regardless of individuals’ migratory status or sex. Together, these results reflect the considerable importance of terrain for bighorn sheep movement and habitat selection^28^. In the case of Sierra bighorn, contiguous preserved habitat across the extent of their seasonal ranges and migratory routes enables animals to maintain proximity to preferred terrain features throughout the migratory season.

High temperatures stand to impact Sierra bighorn through heat stress, while precipitation may lead to terrain instability, wetting leading to hypothermia, or exposure to lightning. Uphill forays by bighorn in this study were associated with lower destination temperatures, presumably related to refuge from heat stress. Heat stress in other ungulates drives similar behavioral responses, leading to selection toward higher elevation and modified daily foraging schedules during hot days^18^. In the eastern Sierra Nevada, high spring temperatures accelerate snowmelt, and, where snowmelt is earliest, the lag between snowmelt timing and green-up timing is greatest^29^. Therefore, higher spring temperatures may cause Sierra bighorn to spend increased time at high elevations while there is still high-quality forage below. Anticipated change in temperature and snowpack dynamics at low- to mid-elevations in the eastern Sierra^30^ may reduce the continuity of low- to high-elevation resource waves^31^, decreasing migratory connectivity and complicating departure schedules of the migrants that live there.

We found a negative effect of precipitation on elevation selection, indicating that storms may drive downhill movements, presumably related to escape from either risk of lightning strike, rockslides, or wind. For example, bighorn sheep in the Convict Creek herd unit undertook downslope movements immediately following a rain event in 2016 and remained at lower elevations for several days thereafter (Supplementary materials S4). While downhill movements in response to storms are known in birds^17^, our study provides evidence of similar responses to storms by ungulates. Notably, the effect of precipitation on selection for elevation was only significantly negative for males, migrants, and in particular slow migrants, indicating that these groups may be more flexible than females and alpine residents in their response to sudden environmental changes. If the frequency of spring and summer storms increases across the Sierra, bighorn sheep may sacrifice foraging opportunities at high elevations in favor of seeking out protected combes and canyons further down mountainsides. Indirect effects of climate change on resource-tracking elevational migrants may thus amplify costs of migration by prompting down- and uphill forays that have both the energetic cost of movement and the opportunity cost of reduced access to forage.

Vertical movements may also facilitate refuge from predation. Because the migratory season of bighorn sheep generally corresponds with lambing, ewes must balance heightened nutritional requirements with selection of habitat that accommodates safe lamb rearing^32,33^, leading to shifts in habitat selection by bighorn ewes after lambing^34^. In the eastern Sierra, mountain lions hunt bighorn sheep with particular success at low elevations^35,36^. Movements toward steep terrain at high elevations may therefore reflect habitat selection for parturition and lamb rearing rather than habitat selection for foraging. In our study, females migrated uphill earlier than males and did not move downslope during storms, possibly reflecting an increased risk of predation at low elevations. Other species of wild sheep also exhibit sex-specific habitat selection, with ewes prioritizing areas that will facilitate lamb growth and survival, and rams prioritizing foraging opportunities at the expense of access to safe terrain^37^. If females are restricted to high-elevation lambing habitat throughout the reproductive season, increasing mismatches between the timing of lambing and the timing of high-quality forage availability may have impacts on long-term population outcomes because Sierra bighorn rely on fat reserves for both successful lambing and overwinter survival^38^.

Model comparison revealed that terrain was the most important class of predictors for step selection by Sierra bighorn. Following terrain, snowpack factors were more important than forage factors for step selection. Notably, however, we analyzed snowpack using a daily 30 m modeled fractional snow cover product^39^, whereas we analyzed forage using 16-day 250 m MOD13Q1 satellite imagery^40^. We suspect the snow model was selected over the green-up model due to the comparatively fine spatial and temporal resolution of the snow product, coupled with the use of a coarse MODIS based NDVI product representing vegetation growth perhaps not completely predictive of forage in sparsely vegetated high alpine landscapes. Combined, these factors could result in the snow product revealing fine-scale landscape phenological variation that is masked at coarser scales^41^. Because snowmelt and plant growth are so tightly linked in alpine systems (e.g. John et al.^29^ and Winkler et al.^42^), we attribute habitat selection for snow properties in this analysis to forage phenology and availability. Indeed, at the coarse level, relative selection was strong for relative NDVI (Fig. 3A), suggesting that Sierra bighorn selected for areas when NDVI peaked at that site.

The 3000 m elevational gradient of the Sierra Nevada generates a broad ecoclimatic window that bighorn sheep can use in response to both short- and long-term abiotic stressors. The Sierra Nevada is expected to experience a decreasing proportion of precipitation falling as snow, and higher snow lines in the coming century^43^. Future reduced snowpack and higher temperatures are expected to be most dramatic at low to mid elevations along the Sierra escarpment and less so in the High Sierra^30,44^, which will likely modify the historic pattern of vegetation green-up, thereby complicating the balance between stress avoidance, forage pursuit, and access to escape terrain. Because the delay between snowmelt and green-up timing is greatest at low elevations where snowmelt is earliest, increasingly early snowmelt at low to mid elevations may lead to a vertical contraction in the range of terrain where plant growth predictably and immediately follows snowmelt. Simultaneously, a higher frequency of hot days may drive bighorn away from areas at a period of peak forage quality. Therefore, if abiotic stress avoidance and forage access are to be maintained, site visitation by bighorn during spring will likely shift toward higher elevations where escape terrain is nearby while maintaining a resource supply that is digestible and nutritious.

An outstanding knowledge gap remains about measurable tradeoffs between ungulate movement and improved access to forage quality or digestibility. To better understand how movement responses to diel and seasonal environmental variation translate into nutritional and energetic outcomes, finer data on bighorn sheep movement and landscape patterns in digestible nutrients are required. Work combining accelerometry and high-resolution remote sensing data could shed light on energy expenditure and intake, particularly if they are paired with measurements of bighorn body condition^38,45^ and plant nutrient concentration. Furthermore, factors such as social information^24^, perception^19^, group cohesion^46^, and intraspecific competition^47^ all play a role in ungulate migrations and will become increasingly important as the Sierra bighorn recovery effort leads to increasing population sizes. Improving inference about individual survival and reproductive capacity, and ultimately carrying capacity at the level of management units, will facilitate conservation of existing Sierra bighorn populations and potentially inform site selection for future reintroductions. For example, candidate reintroduction sites could be prioritized by combining availability of potential refugia from heat stress and storms, in addition to access to escape terrain and high-quality forage. Such a ranking schema would accommodate a broader array of movement tactics by Sierra bighorn across their migratory season.

Whereas long-distance (often latitudinal) migrations allow animals to capitalize on broad-scale variation in resource availability^10,48,49^, elevational migrants (or altitudinal migrants) exploit similar resource dynamics^50,51^ while potentially maintaining a semi-local proximity that lends flexibility to movements between seasonal ranges^13^. An elevational migrant can visit out-of-season ranges, double back over the migratory corridor, and make fine-scale movement adjustments during the migration season that could not be achieved by a migrant whose seasonal ranges are separated by hundreds of kilometers^52^. Our work indicates that bighorn sheep make movement decisions during the migration season in response to environmental stressors while seeking out preferred fine-scale terrain features and simultaneously maintaining coarser responses to landscape phenology. This suggests that movement decisions may be guided by static landscape variability (terrain) and then further informed by dynamic landscape variability, which can emerge in the form of a resource (forage access) or a stressor (storms or high temperatures). For elevational migrants, dynamic landscape variability may be mitigated by moving a few hundred meters up- or downhill, so a longer migration duration may arise from fine-scale temporal variation in selection for elevation. Together, these findings highlight the importance of examining nested scales of movement and interrelated landscape dynamics in migratory species faced with multifaceted environmental change.

## Methods

### Study system

Sierra Nevada bighorn sheep are alpine specialists and partial, facultative, elevational migrants in the Sierra Nevada mountains of California (Spitz et al.^21^, Berger et al.^19^; Fig. 1). The gradient from the base of the Sierra’s escarpment to its highest peaks often exceeds 2 km elevation and features a predictable elevational delay in plant phenology^29^. Individuals that undergo uphill spring displacement typically follow one of two migratory patterns^13^: In undistracted migrations, individuals undertake a single, uphill trip, departing from low-elevation winter range and settling on high-elevation summer range. In vacillating migrations, individuals undertake multiple up-and-down movements over a period of days or weeks before settling on high-elevation summer range^13^. Sierra bighorn occupy 14 “herd units”, a spatial delineation used for conservation metrics and management strategies, and which approximately represent discrete bighorn populations^53^.

### Movement data

Sierra bighorn individuals were fitted with GPS collars (various models from Advanced Telemetry Systems, North Star Science and Tech LLC, LOTEK Engineering Ltd., Televilt, VECTRONIC Aerospace GmbH, Followit, and Sirtrack LTD; described in^54^) during spring and fall capture seasons (March and October) between 2002 and 2022. Animal handling was done under veterinarian supervision and approved under the California Department of Fish and Wildlife Animal Welfare Policy (2017-02). In total, 311 unique individuals were tracked for a total of 702 animal-years, with an average of 35 animals per year. Collars were deployed in all 14 herd units, spanning the full latitudinal, longitudinal, and elevational range occupied by the species. Collars were programmed to collect GPS locations at a minimum frequency of 1 fix per 12 h.

### Habitat covariates

USGS 3DEP 10 m National Map elevation data were acquired via Google Earth Engine^55,56^. Slope and aspect were calculated using the 4-neighbor rule (comparing the focal pixel with adjacent rook moves). Bighorn sheep strongly rely on being on or near escape terrain (rugged terrain where they are relatively safe from predators); as is typically done, escape terrain was classified using a 30° slope threshold (sensu^57^). Distance from escape terrain was calculated using the fasterraster v.0.6.0 R plugin for QGIS v.3.22^58,59^.

Seasonal and spatial variability in vegetation production were summarized using MOD13Q1 NDVI at 250 m resolution^40^ following^60,61^. For each pixel, NDVI was rescaled by transforming the bottom 2.5 percentile and top 97.5 percentile to 0 and 1, respectively. Negative values were raised to 0. The rescaled time series was smoothed using a moving median window (width = 3; see^61^). The smoothed time series was fit to a double logistic function following the form:

1 $$ NDVI = \frac{1}{{1 + {\text{exp}}\left( {\frac{xmidS - x}{{scalS}}} \right)}} - \frac{1}{{1 + {\text{exp}}\left( {\frac{xmidA - x}{{scalA}}} \right)}} $$

where x is the ordinal day of year, xmidS and xmidA are the ordinal days of green-up and senescence inflection points respectively, and scalS and scalA are scaling parameters describing the rate of green-up and senescence, respectively^60^. The pixel-specific parameterization of Eq. (1) was used to index seasonal variability in forage production (“relative NDVI”). Relative NDVI has values close to 1 when a pixel is at its peak level of biomass production for the year, and values close to 0 when a pixel is at its lowest levels of production. Forage production (“absolute NDVI”) was indexed by un-scaling the parameterized predictions of Eq. (1) using the 2.5 and 97.5 percentile scaling parameters from the original raw NDVI transformation. In this way, areas with dense vegetation have values closer to 1 and areas with sparse vegetation have values closer to 0. Green-up timing was measured using the pixel-specific xmidS parameter, which is the date during which relative NDVI increases at the fastest rate, and days since peak green-up was calculated as the difference between a given date and the date of green-up timing.

A modeled daily snow dataset was used to index fractional snow cover (FSC) and snowmelt timing^39^. This dataset was generated using a data fusion and machine learning approach that combines Landsat 5, Landsat 7, and spatially and temporally complete (STC) MODIS satellite imagery^62^ to generate daily FSC estimates at 30 m resolution. Both the MODIS STC algorithm and the Landsat 5 and 7 FSC rely on spectral mixture analysis^63^ to estimate FSC which is considered more accurate than index-based methods for estimating snow cover^64–67^. The 30 m FSC modeled dataset was developed using 5% of inputs as training and 95% as validation, and showed a 97% accuracy with 1% bias. To increase confidence in the accuracy of FSC, the product was additionally validated using snow cover data from an independent in situ sensor network^29,68^. The validation reveals strong concurrence among the modeled FSC dataset and point estimates of snow cover throughout the range of Sierra bighorn (Supplementary materials S1). To summarize seasonal snow cover variability, FSC was fit to Eq. (1) above. Snow cover fraction (with a range of 0–100%) was used in place of NDVI and the curve was accordingly fit on a [0,100] interval. Year was offset to start on August 15 and end on August 14 so that each FSC time series began at a summertime baseline (0% snow cover), accumulated to a wintertime maximum, and then deteriorated during the melt season back to 0. Snowmelt timing was measured using the pixel-specific xmidA parameter, which is the date at which the fitted FSC decreases at the fastest rate, and days since peak snowmelt was calculated as the difference between a given date and the date of snowmelt timing.

Daily temperatures and precipitation were extracted from the DAYMET V4 dataset at 1 km resolution^69^. Heat can be physiologically taxing for alpine ungulates that are adapted for cold temperatures, and may drive uphill movements that mitigate heat stress experienced at lower elevations at the cost of foraging opportunities^18,70^. We used daily maximum temperature to index potential thermal stress across the eastern Sierra. Precipitation is infrequent in the Sierra outside winter, but is often associated with high winds and lightning, and causes terrain in the alpine zone to become wet and particularly unstable^71^. Rock slides have been attributed to mortality in several alpine caprine species, including ibex^72^ and Dall sheep^73^, and mountain sheep have been observed to react quickly to warning signs of rockfall^28^. We used water-equivalent total daily precipitation (mm) to index rainfall variability in the region.

### Migration classification

Seasonal elevation use was determined by extracting elevation from the 3DEP National Map using bighorn GPS location data. Migrants were classified using the migrateR package^74^. MigrateR uses an elevational analogue for measuring net squared displacement, and classifies individuals as “resident”, “disperser”, or “migrant” based on a model comparison approach between a consistent position through time, a single upward movement across time, or an up-and-down redistribution through time. We used minimum thresholds of 500 m between elevational ranges and 21 days spent on each seasonal range when classifying individuals as residents, dispersers, and migrants based on previous work in this system^22,74^. Because the focus of this study was on uphill spring migration, any uphill dispersers were combined into the migrant class. Migration timing was quantified using the theta parameter from the elevational net squared displacement curves, which indexes the midpoint of the individual’s departure movement. Migration rate was quantified using the phi parameter; migrants were classified as “fast migrants” if the migration lasted less than 1 week and “slow migrants” if the migration lasted at least 1 week.

### Step selection modeling

Habitat selection was examined using integrated step-selection analysis (iSSA^75^). We conducted a single (population-level) iSSA (rather than multiple individual iSSAs) because minimal endpoint variance in predictor variables constrained our ability to resolve movement processes for animals that used short step lengths relative to coarse-resolution remote sensing data. A population-level model allowed inclusion of a greater number of individuals and testing of a greater number of candidate movement drivers simultaneously. To maintain sampling consistency across individuals, the 21 days centered on each migrant’s migratory window was used for the analysis. For residents, the 3 weeks centered on the mean migration timing of that individual’s herd unit in that year was used. If no migrants were detected in a resident’s herd unit in a given year, the resident was excluded from the analysis. Our model included equal numbers of GPS observations from each individual in order to avoid bias in the model design.

In cases where the GPS fix rate was more frequent than 12 h, relocation data were temporally rarified to a 12-h frequency. Each resampled fix was treated as a startpoint, with the following fix treated as a used endpoint. Thirty random destinations were used as available endpoints. Endpoints were drawn from gamma and von Mises distributions fit to the population’s step length and turning angle history, respectively^75–77^.

Environmental covariates were extracted at all start- and endpoints. Terrain features were treated as fixed across time. Snowmelt timing and green-up timing were fixed across time within years, while FSC, distance from snow, relative NDVI, absolute NDVI, maximum temperature, and total precipitation all varied daily. Elevation, terrain slope, and temperature were scaled using z-scores across the full extracted dataset to aid in model fitting. Aspect was cosine-transformed such that north-facing slopes were 1 and south-facing slopes were − 1. Precipitation, distance from escape terrain, and distance from snow were transformed using log(value + 1) to accommodate 0s. Hot days were identified as temperatures exceeding the 75th percentile of daily temperatures at the level of the individual bighorn-year.

Drivers of habitat selection during migration movements were evaluated by fitting an integrated step-selection function to the data using conditional logistic regression^78,79^ with case (observed endpoint = 1; randomly sampled endpoint = 0) as the response variable, habitat covariates as candidate predictor variables, and step ID as the stratification variable. Two model families were built: First, a complete movement model included fixed endpoint conditions to identify drivers of habitat selection (i.e., a movement outcome). Endpoints included terrain parameters (elevation, slope, aspect, and distance to escape terrain), daily weather parameters (high temperatures and cumulative precipitation), snow parameters (FSC, distance to snow, and days since peak snowmelt), and forage parameters (absolute NDVI, relative NDVI, and days since peak green-up). Interactions were included between endpoint elevation and the binary statuses of “hot day” and “raining” at the beginning of movement. The model was cross-validated using a fivefold partition with 100 repetitions (Supplementary materials S2^80,81^). In the second family of models, we sought to explore patterns of variation in movement behaviors between sex and migratory strategy in order to test two hypotheses: Step selection during the migratory season will differ between male and female bighorn sheep if competing life history demands outweigh potential benefits of alternative movement options. For example, females may be more constrained to high elevations, in spite of high-quality foraging opportunities at lower elevations, if predation risk at low elevations affects lambing success. And, step selection will differ according to migratory strategy if responsiveness to environmental variability underlies migratory decision-making. Whereas “fast” migrants may move from distinct low- to high-elevation ranges in one go, “slow” migrants that undertake a series of vertical forays could ostensibly respond to a gamut of seasonal pressures while maintaining access to foraging opportunities. All of the same predictor variables were used as in the overall population-level iSSA.

All statistical analyses were done using R version 4.1.2^82^.

### Ethical approval

Bighorn sheep collaring activities were approved under University of Montana Institutional Animal Care and Use Committee (024-07MHWB-071807 and 46-11), California Department of Fish and Wildlife Animal Welfare Policy (Sierra Nevada Bighorn Sheep Capture Plan 2006-10-2018-10), and United States Fish and Wildlife Service Recovery Permit (#TE050122-6 to TRS).

### Supplementary Information

Supplementary Information.

## Data Availability

Workflow code and input data for iSSF models are available at https://github.com/JepsonNomad/finescale_migration. GPS data are archived in the Sierra Nevada Bighorn Sheep Database of the California Department of Fish and Wildlife. Because of the sensitive nature of biological data for endangered species, data requests are handled on a case-by-case basis and data requests can be submitted to asksnbs@wildlife.ca.gov.
