# Supplementary material for: Pursuit and escape drive fine-scale movement variation during migration in a temperate alpine ungulate
Source: Sci Rep. 2024 Jul 2;14:15068. doi: 10.1038/s41598-024-65948-8 (PMC11219842; doi:10.1038/s41598-024-65948-8)

Supplementary materials S1. Contingency table of snow observations in a time-lapse camera network and binarized snow predictions built using the modeled snow dataset described in Rittger et al 2021. Notably, time-lapse imagery was collected at a finer scale and therefore some disparity between the products is to be expected. Despite differences in spatial resolution, the two data sources show overwhelming agreement (50420 observations; agreement rate = 90.50%; rate of commission = 2.46%; rate of omission = 7.03%).


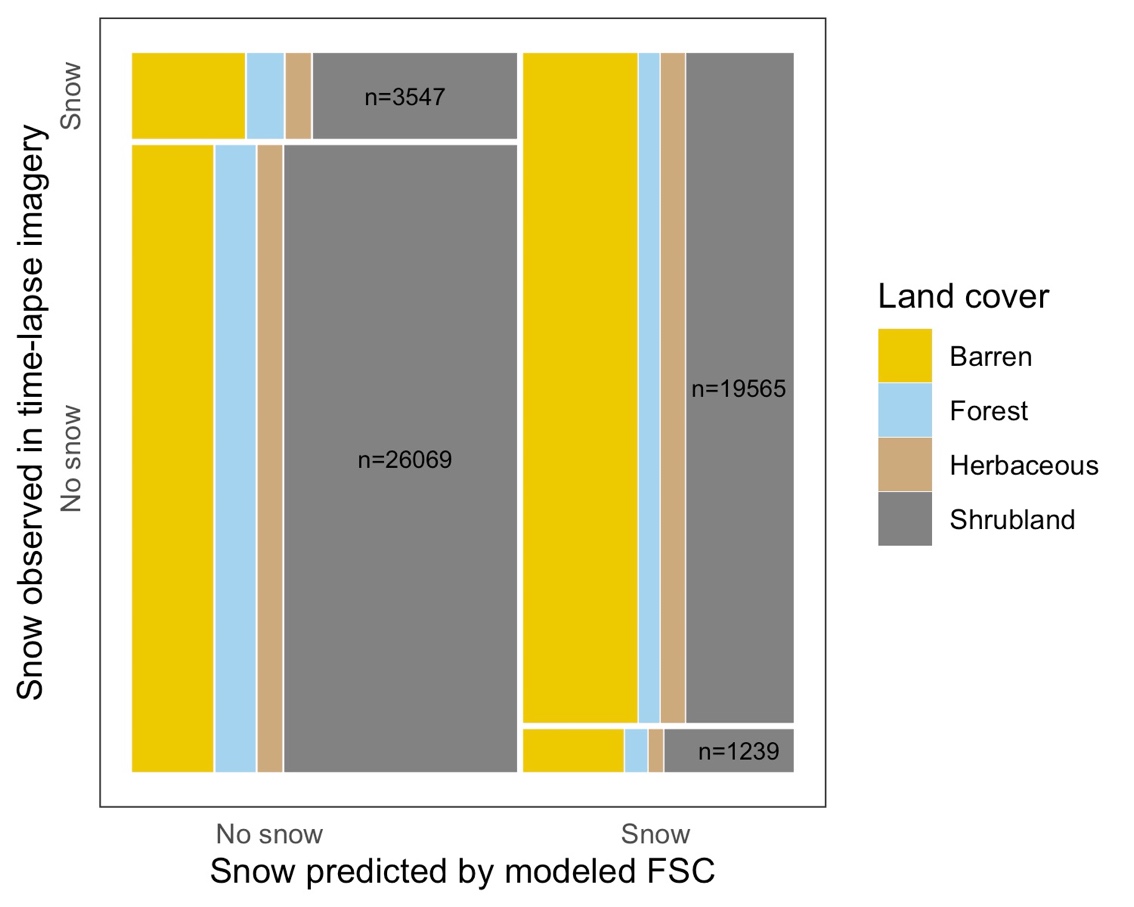


Supplementary materials S2. Cross-validation of population-level integrated step selection analysis (iSSA) using k-fold cross-validation with 5 subsets and 100 repetitions; for details refer to (Latombe et al. 2014, Basille 2015). Validation results are expressed in terms of correlation among the k folds. Correlation is close to 1 when subset model predictions agree; correlation is 0 when there is no relationship between models. Models were generated from random subsets of the observed data in Group = Observed and with random results when Group = Random. Mean correlation for the observed group was 0.93±0.02; and for the random group was -0.015±0.19.


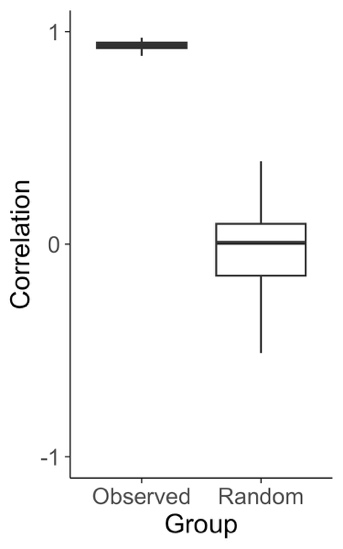


Supplementary materials S3. Table of migration strategy and speed by herd unit. Individual-years calculated as the total number of individuals falling into each of the classes each year across the full dataset.


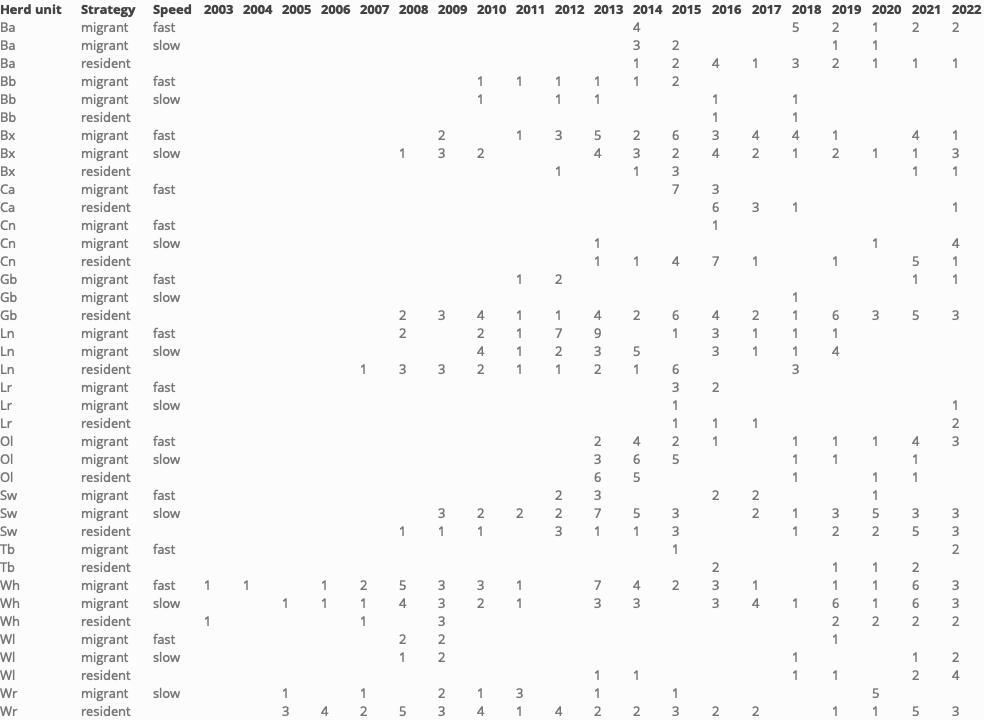


Supplementary materials S4

Three bighorn sheep (indicated with differing line types) in the Convict Creek herd unit selected lower-elevation terrain following a period of heavy rain in early June, 2016 (rain events indicated with grey vertical lines).


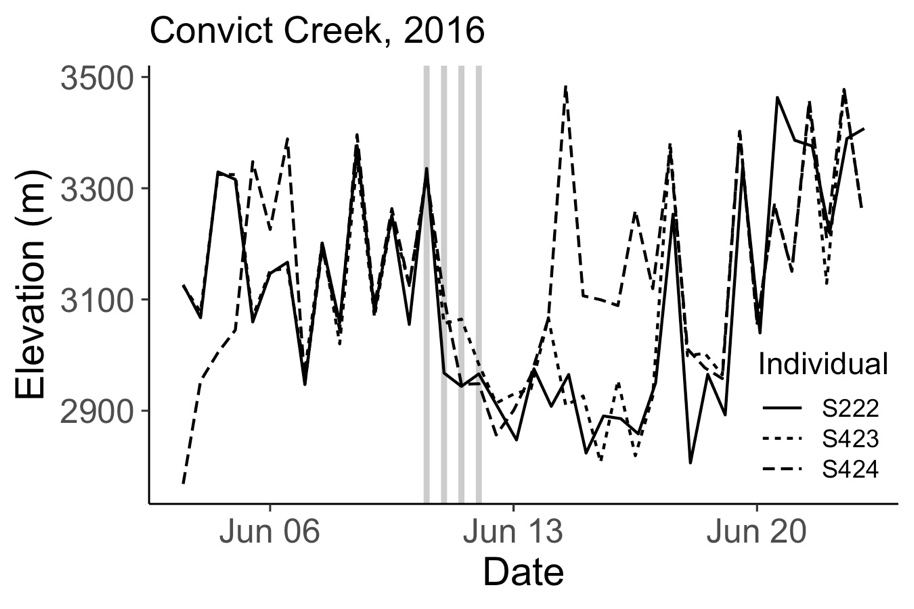

Supplement: Supplementary file 1 — Supplementary Information. [file 41598_2024_65948_MOESM1_ESM.docx]
